# Torticolis de l'enfant révélant une tumeur médullaire

**DOI:** 10.11604/pamj.2015.21.26.6944

**Published:** 2015-05-12

**Authors:** Sonia Jemni, Samia Frioui

**Affiliations:** 1 Service de Médecine Physique et de Réadaptation Fonctionnelle, CHU Sahloul, Faculté de Médecine « Ibn El Jazzar », Sousse, Tunisie

**Keywords:** Torticolis, enfant, tumeur, torticollis, child, tumor

## Image en medicine

Le torticolis est une contracture d'un ou de plusieurs muscle(s) du cou. Il se manifeste par un tableau plus ou moins douloureux associant inclinaison de la tête du côté atteint, translation et rotation du côté sain. Les étiologies sont nombreuses. Leur gravité est variable, incluant des pathologies musculaires, traumatiques, inflammatoires, infectieuses et tumorales. La majorité de ces torticolis sont bénins mais ne dispensent pas d'un examen complet qui permet d'éliminer les causes rares et graves. Nous rapportons le cas d'un enfant âgé de 10 ans, qui consultait pour un torticolis douloureux évoluant depuis 1 mois, sans notion de fièvre, ni d'infection ORL ou de contexte traumatique évident. L'examen clinique montrait une importante contracture du muscle sterno-cléido-mastoïdien gauche avec une mobilité cervicale très limitée et douloureuse. L'examen neurologique n'a pas révélé d'anomalies des réflexes ou d'atteinte des paires crâniennes. L'enfant a été revu quinze jours après cette consultation avec persistance de la même symptomatologie. Malgré un examen neurologique normal et devant la persistance de la symptomatologie, une Imagerie par résonance magnétique cérébrale et cervicale a été demandée. Cette dernière a montré un processus tumoral tissulaire avec composante kystique supérieure de développement intra médullaire étendu sur environ 8 cm de hauteur de C1 jusqu'à C7 réalisant une grosse moelle occupant la totalité du canal rachidien au niveau cervical. L'aspect IRM de cette lésion évoquait en premier lieu un astrocytome. La décision thérapeutique était l'abstention chirurgicale vu le risque certain de tétraplégie post opératoire et que l'enfant ne présentait pas encore de déficit neurologique.

**Figure 1:**
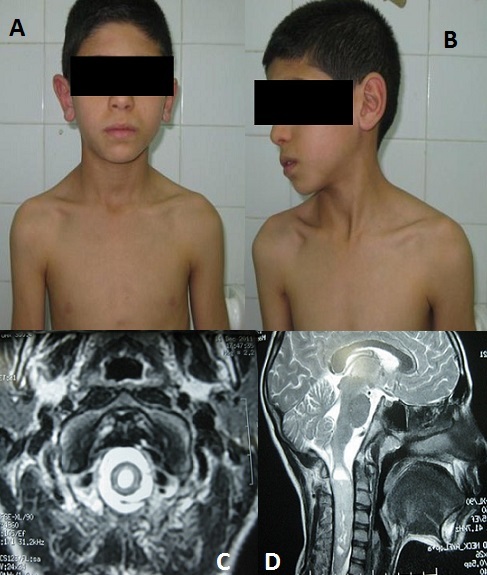
(A) photographie de face montrant la contracture des sterno-cléido-mastoïdiens; (B) photographie en rotation droite de la tête montrant la contracture des sterno-cléido-mastoïdiens; (C) IRM en coupe transversale du rachis cervical: un processus tumoral tissulaire avec composante kystique, intra médullaire réalisant une grosse moelle occupant la totalité du canal rachidien au niveau cervical; (D) IRM en coupe sagittale du rachis cervical: un processus tumoral tissulaire avec composante kystique supérieure de développement intra médullaire étendu sur environ 8 cm de hauteur de C1 jusqu’à C7

